# Target prices for mass production of tyrosine kinase inhibitors for global cancer treatment

**DOI:** 10.1136/bmjopen-2015-009586

**Published:** 2016-01-27

**Authors:** Andrew Hill, Dzintars Gotham, Joseph Fortunak, Jonathan Meldrum, Isabelle Erbacher, Manuel Martin, Haitham Shoman, Jacob Levi, William G Powderly, Mark Bower

**Affiliations:** 1 Department of Pharmacology and Therapeutics, University of Liverpool, Liverpool, UK; 2 Faculty of Medicine, Imperial College London, London, UK; 3 Chemistry and Pharmaceutical Sciences, Howard University, Washington DC, USA; 4 Faculty of Medical Sciences, University College London, London, UK; 5 Institute for Public Health, Washington University in St. Louis, St. Louis, Missouri, USA; 6 National Centre for HIV Malignancy, Chelsea & Westminster Hospital, London, UK

**Keywords:** HEALTH ECONOMICS, ONCOLOGY, PUBLIC HEALTH, THERAPEUTICS

## Abstract

**Objective:**

To calculate sustainable generic prices for 4 tyrosine kinase inhibitors (TKIs).

**Background:**

TKIs have proven survival benefits in the treatment of several cancers, including chronic myeloid leukaemia, breast, liver, renal and lung cancer. However, current high prices are a barrier to treatment. Mass production of low-cost generic antiretrovirals has led to over 13 million people being on HIV/AIDS treatment worldwide. This analysis estimates target prices for generic TKIs, assuming similar methods of mass production.

**Methods:**

Four TKIs with patent expiry dates in the next 5 years were selected for analysis: imatinib, erlotinib, lapatinib and sorafenib. Chemistry, dosing, published data on per-kilogram pricing for commercial transactions of active pharmaceutical ingredient (API), and quotes from manufacturers were used to estimate costs of production. Analysis included costs of excipients, formulation, packaging, shipping and a 50% profit margin. Target prices were compared with current prices. Global numbers of patients eligible for treatment with each TKI were estimated.

**Results:**

API costs per kg were $347–$746 for imatinib, $2470 for erlotinib, $4671 for lapatinib, and $3000 for sorafenib. Basing on annual dose requirements, costs of formulation/packaging and a 50% profit margin, target generic prices per person-year were $128–$216 for imatinib, $240 for erlotinib, $1450 for sorafenib, and $4020 for lapatinib. Over 1 million people would be newly eligible to start treatment with these TKIs annually.

**Conclusions:**

Mass generic production of several TKIs could achieve treatment prices in the range of $128–$4020 per person-year, versus current US prices of $75161–$139 138. Generic TKIs could allow significant savings and scaling-up of treatment globally, for over 1 million eligible patients.

**Strengths and limitations of this study**

- This study calculated estimated generic prices for four tyrosine kinase inhibitors using an algorithm based on publicly available data on completed sales of the pharmaceutical ingredients.
- Publicly available data were used to calculate the global number of people eligible for treatment, as well as to present a global price overview, for each medicine.
- The estimation methods are limited by the assumption of absence of intellectual property and other trade barriers, and the assumption of robust demand volume and market competition for these medicines.
- The methods used to estimate the global number eligible for treatment with the medicines are limited by sparse data on cancer subtype epidemiology—the effect is likely to be one of underestimation.

## Introduction

Worldwide, there were 8.2 million deaths due to cancer in 2012,^1^ and incidence is expected to rise by 70% over the next 20 years.^2^ The majority of cancer cases and deaths occur in Africa, Asia, Central and South America.^2^ Fatality rates are much higher in low-income and middle-income countries (LMICs). For all cancers, the case fatality rate is 74.5% in low-income countries, compared to 46.3% in high-income countries.^3^

Tyrosine kinase inhibitors (TKIs) target tumour cells by interfering with signalling pathways that are involved in cell growth and division.^4^ Imatinib mesylate is licensed as first-line treatment for adults with chronic-phase Philadelphia-chromosome-positive (Ph+) chronic myeloid leukaemia (CML), and for the management of gastrointestinal stromal tumours (GIST), and as salvage therapy for Ph+ acute lymphoblastic lymphoma.^5^ Erlotinib is licensed as a first-line treatment of locally advanced or metastatic non-small cell lung cancer (NSCLC) with activating epidermal growth factor receptor mutations.^6^ Sorafenib is licensed as a second-line treatment for renal cell carcinoma (RCC) and unresectable hepatocellular carcinoma (HCC).^7^ Lapatinib is licensed for advanced HER2-positive breast cancer.^8^ There were no TKIs in WHO's Model List of Essential Medicines (EML) until the recently published 19th edition, in which the only TKI is imatinib,^9^ ^10^ despite strong evidence for the efficacy of other TKIs. NGOs have highlighted that the high prices of medicines pose a potential obstacle to their inclusion,^11^ as comparative cost-effectiveness is a criterion for addition to the WHO EML.^12^ The low number of TKIs on the WHO EML is reflected in national Essential Medicines Lists. Over 75% of national EMLs in all regions except Europe do not include any tyrosine kinase inhibitors, and in nearly all LMICs, public procurement is based on national EMLs.^13^ It has been estimated that only 15% of patients in LMICs in Southeast Asia have access to an index of cancer medicines, including erlotinib and sorafenib.^14^ High prices act as a barrier to access also in high-income countries. For example, in the UK, sorafenib is not available in the NHS due to insufficient cost-effectiveness.^15^ The impact of this lack of access on patients has been widely documented.^16–18^ The high prices of leukaemia drugs have been strongly criticised by a large group of experts, who have suggested they conflict with fulfilling the Hippocratic Oath.^19^

The price-reducing effect of generic competition can transform how diseases are treated. In the field of HIV/AIDS medicines, generic competition was encouraged by resource allocation for their purchase and the use of flexibilities in trade law allowing the importation of generics where normally importation would have been prevented by patent protection. The 99% reduction in the prices of antiretrovirals following generic competition, from $10 000 per person per year down to $100 has been a key factor in the expansion of antiretroviral treatment to over 13 million people in 2014.^20^ ^21^ Similar analyses of minimum prices have been performed for hepatitis C drugs,^22^ and for the hepatitis B treatment entecavir.^23^ This paper estimates target prices for generic TKIs that could be achieved when their patent terms expire within the next 5 years, or when patents no longer form a barrier to generic entry otherwise—for example by licensing to generic manufacturers.

## Methods

We focus on four TKIs with anticipated patent expiry dates within the next 5 years. The chemical structures and excipient contents for all TKIs were gathered from prescribing information published by the originator companies (see online supplementary appendix 1). For each TKI, chemical structures, dosing and published data on per-kilogram pricing for the active pharmaceutical ingredient (API) were reviewed. Analysis included costs of excipients, formulation, packaging, shipping and a 50% profit margin. Results were validated by independent estimates from a single large generic company.

### Calculation of treatment cost

We derived target prices using an algorithm based on per-kilogram prices of the APIs, previously used in analyses of drugs for hepatitis C and B.^22^ ^23^ Current manufacturers of API were contacted to request quotes for price per kilogram, and export data for India were reviewed for 2014 and early 2015 to estimate a reasonable lower price for the APIs.^24^

**Figure 1:**
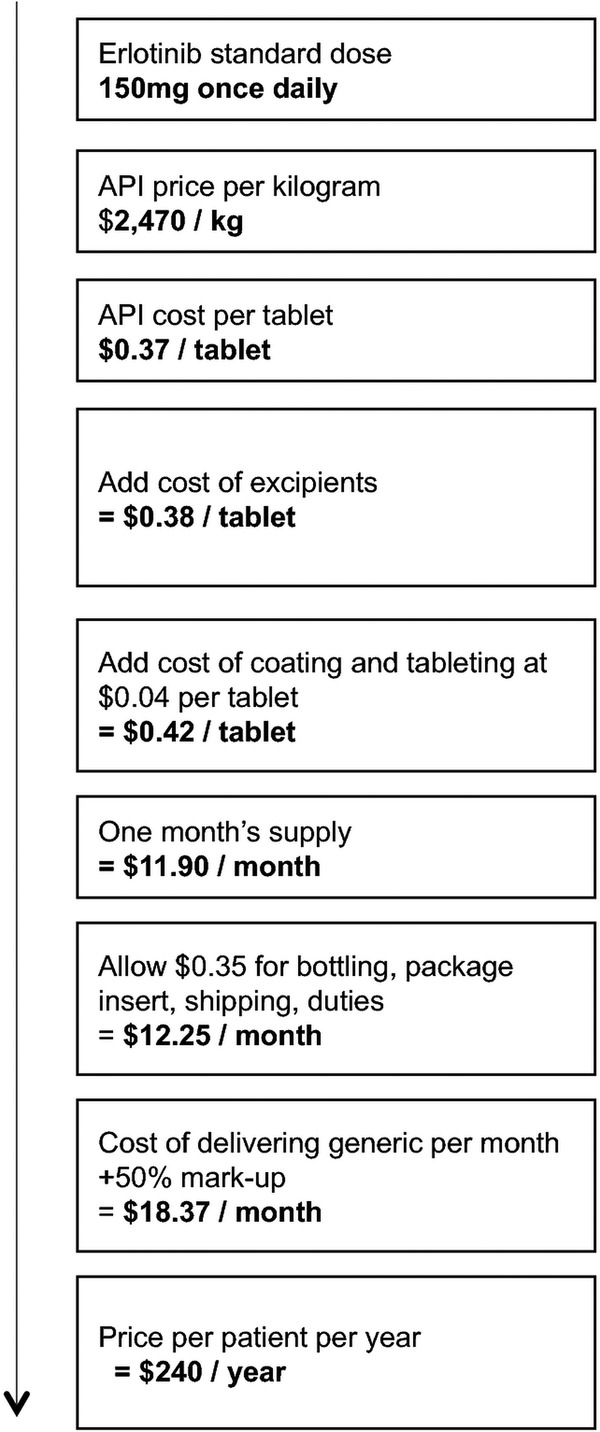
Cost estimation flow chart for erlotinib. API, active pharmaceutical ingredient.

Calculations for all TKIs analysed are shown in table 1, and the target price calculation for erlotinib is displayed as a flowchart in figure 1 as an example of the algorithm used. The dose of erlotinib is 150 mg once daily, so 1 year's supply of the drug would require 55 g of the API. One kilogram of erlotinib API was estimated to cost $2470. Annual dosing regimens were combined with API prices to yield the per-tablet cost of API ($0.37). We added conservative estimates for the costs of excipients and tableting and multiply by 30 to yield monthly cost of production ($11.90/month). The prices of excipients were incorporated into the target price by assuming that all of the non-API mass of the tablet is made up of the most expensive excipient, and that the total weight of the tablet is five times the weight of API alone. To this cost estimate, we added costs of shipping and duties at $0.35/month, assuming one bottle delivered to the patient every month ($12.25/month). Costs estimated for these components are conservative and would represent a relatively inefficient manufacturing process. Last, we added a 50% mark-up to this cost of production to estimate a target price that would be profitable and sustainable, to encourage market entry and competition among generic producers ($18.37/month). We divided this price by 28 and multiplied by 365 to give a target price per patient-year ($240/year).

**Table 1:** Assumptions and calculations of target prices

Medicine	Imatinib	Erlotinib	Sorafenib	Lapatinib
API per tablet	400 mg	150 mg	200 mg	250 mg
Tablets per month	28	28	112	168
API price per kilogram	$347–$746	$2470	$3000	$4671
API cost per tablet	$0.14–$0.30	$0.37	$0.60	$1.17
Add cost of excipients and formulation	$0.18–$0.34	$0.38	$0.62	$1.18
Add cost of tableting	$0.22–$0.38	$0.42	$0.66	$1.22
Cost per month	$6.22–$10.68	$11.90	$73.83	$205.26
Add cost of bottle, packaging, shipping, duties	$6.57–$11.03	$12.25	$74.18	$205.61
Add 50% mark-up	$9.85–$16.55	$18.37	$111.27	$308.41
Target price per year	$128–$216	$240	$1450	$4020

The prices of excipients used for each TKI are given in text, but not shown in table.

API, active pharmaceutical ingredient; TKI, tyrosine kinase inhibitor.

### Patent coverage and global prices

Estimated patent expiry dates for the USA and EU were gathered from originator company reports (see online supplementary appendix 2). The patent statuses of the TKIs in India were reviewed.

Prices for the chosen TKIs were identified in 12 countries, using national databases and online price comparison tools (see online supplementary appendix 3). In all cases, the lowest available price per pill was used for comparison. Where pricing information for a medicine was not found for a country, no bar is displayed.

### Incidence of cancers and volume demand

Using published figures of the epidemiology of cancers for which the chosen TKIs are indicated, we conservatively estimated annual volume of demand, in terms of the number of people newly eligible for treatment per year. We estimated the incidence of all cancers treated with the TKIs analysed, including renal cell carcinoma, hepatocellular carcinoma, thyroid carcinoma, chronic myeloid leukaemia, acute lymphoblastic leukaemia, pancreatic cancer, non-small cell lung cancer and breast cancer. The annual number eligible is multiplied by the annual requirement of API in grams, per patient, to give annual volume demand. Our assumptions and estimates are presented in table 2, and references used are given in online supplementary appendix 4.

**Table 2:** Indications, dosing, originator company and patent expiry dates for selected TKIs

Medicine	Indication(s)*	Dose(s)*	Originator company	Expiry of term for base compound patent†		Target price per patient per year
				USA	EU	
Imatinib (Glivec/Gleevec)	Chronic myeloid leukaemia	400 mg QD	Novartis	2015	2016	$128–$216
Erlotinib (Tarceva)	Non-small cell lung cancer (locally advanced or metastatic)	150 mg QD	Roche	2018	2020	$240
Sorafenib (Nexavar)	Renal cell carcinoma, Hepatocellular carcinoma	400 mg BID	Bayer and Onyx Pharmaceuticals	2020	2020	$1450
Lapatinib (Tyverb/Tykerb)	Advanced breast cancer	1500 mg QD	Novartis	2020	2023	$4020

*References in online supplementary appendix 1.

†References for patent expiry dates are given in online supplementary appendix 2 and assume no supplementary patent term extensions.

BID, two times a day; QD, daily; TKI, tyrosine kinase inhibitor.

Incidence data for International Classification of Diseases (ICD) 10 categories was obtained from *Globocan 2012*,^1^ and incidence of specific cancer types was estimated from these figures using data from other studies on the proportion of cases of the cancer subtype within the ICD10 group. For example, renal cell carcinoma is included in the ICD10 category ‘kidney cancer’, and represents 85% of incidence in this category. In breast cancer, data was only available for female incidence.

In our estimates of the number globally eligible for treatment, we included published data on the proportion of cases that are receptor/chromosome positive, relapsed/refractory to treatment, and advanced/metastatic at presentation. Owing to the lack of similar data for LMICs, these estimates are largely based on data from high-income countries (HICs); where figures were available for both, these figures were combined to estimate global incidence.

Our estimates assumed full access to all interventions indicated before use of TKIs, including surgery, radiotherapy and chemotherapy. We do not include measures of access in our assumptions; where patients do not have access to these interventions, TKIs may provide the best available treatment due to low cost, potentially increasing the eligible population. In addition, data from HICs for the proportion of cases that are advanced/metastatic at presentation is likely to underestimate the proportion in countries with reduced access to healthcare services and health information. Our estimates of the global eligible population are thus conservative.

## Results

### Chemical descriptions and calculated target prices

**Figure 2:**
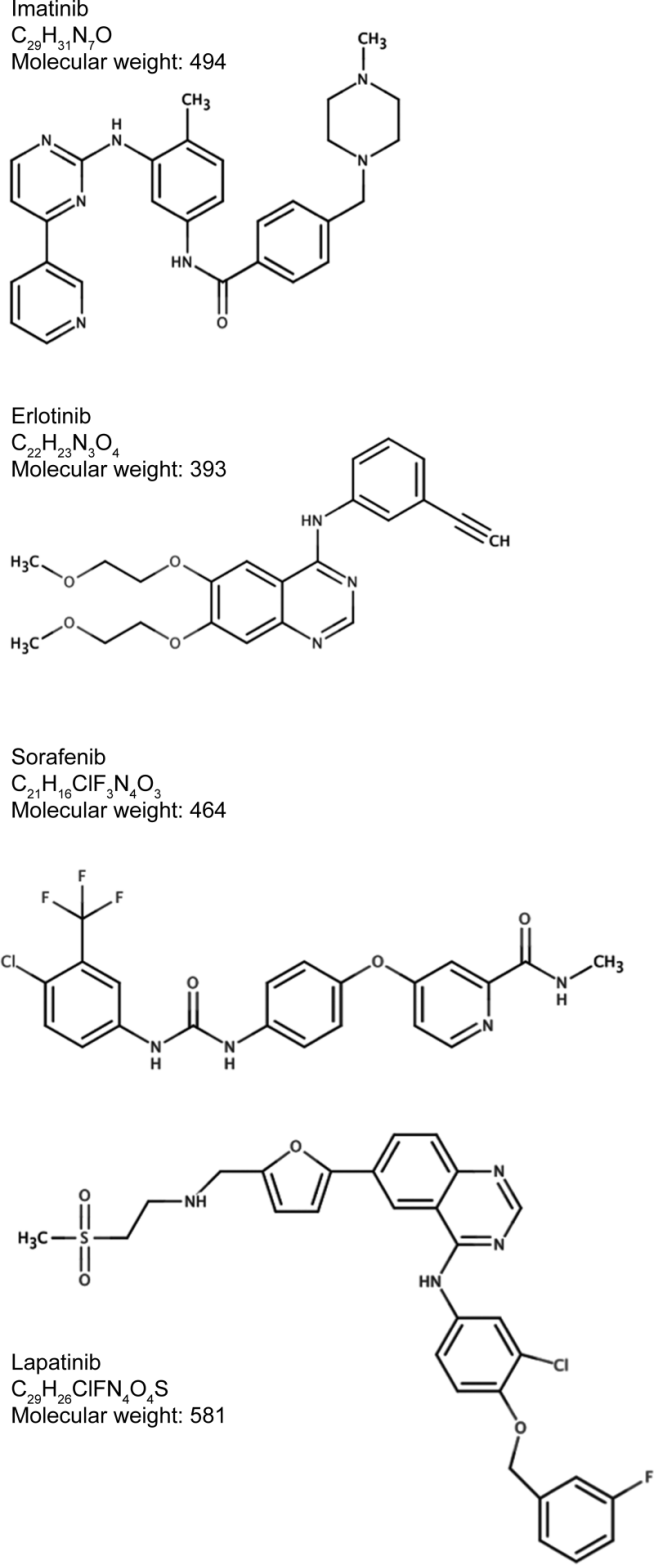
Chemical structures, formulae and molecular weights.

The chemical structures of the TKIs are shown in figure 2. Calculations of treatment cost are shown in table 1. The price of API for imatinib, erlotinib and lapatinib have been estimated primarily using data on exports from India,^24^ while the API price for sorafenib was obtained by personal communication with a large generic manufacturer.

#### Imatinib

The standard dose for imatinib is 400 mg daily, equivalent to an API requirement of 146 g per person-year. Prices of exported imatinib have decreased dramatically over the last 5 years, as multiple generic manufacturers compete, and as manufacturing processes are optimised (data not shown). Nevertheless, a wider distribution of stable prices is seen in imatinib API than for the other drugs. For imatinib, we therefore present a range of estimated target prices.

There is already a significant demand in volume for imatinib. There are multiple suppliers of API, and there are alternative processes for which patent applications have been filed. API is sold at a wide range of prices to different markets: distinct markets for the API exist, for which the pricing may be as low as $340/kg. In 2014, 68 kg of imatinib API were shipped for $340–$347/kg. A market of $340–$1000/kg exists for Argentina, Ecuador, Bangladesh, Singapore, Mexico and the USA; this market represents an approximate total volume of 750 kg of API exported from India in 2014 in 15 shipments. In medium-tiered pricing markets, we see a range of $1000–$2000/kg for the API including countries UAE, Jordan and Bangladesh, representing an approximate export volume of 840 kg of API in the last year from India. In high-tiered pricing markets, API is exported from India to the UAE, Israel, Canada, Iran and the USA, with a price range of $2000–$5000/kg, and an approximate volume of 4.5 tonnes in the last year from India.

We have estimated a range of target prices based on the robust low-tier market, using an API price of $347/kg for the lower estimate, and $746/kg for the higher estimate (weighted average within the $340–$1000/kg market). The most expensive excipient in imatinib mesylate is crospovidone (median price $27/kg)*.* This yields a per-year target price of $128–$216.

#### Erlotinib

The standard dose for erlotinib is 150 mg daily, equivalent to an API requirement of 55 g per patient per year. Erlotinib API exports from India showed a lowest price of $2470/kg in 2014. The most expensive excipient used is hypromellose (median price $24/kg). This yields a per-year target price of $240.

#### Sorafenib

The standard dose for sorafenib is 400 mg twice daily, equivalent to an API requirement of 292 g per patient per year. Sorafenib API exports from India showed a lowest price of $7472 per kilogram in 2014, with a low volume of total shipments. However, we received a quote of $3000/kg from a large Indian generics company, which we used for our target price estimate. The most expensive excipient used is hypromellose (median price $24/kg). This yields a per-year target price of $1450.

#### Lapatinib

The standard dose for lapatinib is 1500 mg once daily, equivalent to an API requirement of 548 g per patient per year. Lapatinib API was exported from India twice in 2014, with a mean price of $4674/kg. The most expensive excipient used in lapatinib ditosylate is povidone (median price $14/kg). This yields a per-year target price of $4020.

### Patent expiry

Expiry dates of patent protection for the TKIs surveyed are presented in table 2 and references are given in online supplementary appendix 2. Basic patent protection for imatinib mesylate will expire in 2015 (USA) and 2016 (EU). For erlotinib—2018 (USA) and 2020 (EU). For sorafenib—in 2020 (USA and EU). For lapatinib—in 2020 (USA) and 2023 (EU).

Imatinib and sorafenib are not under patent protection in India. Lapatinib is under patent protection in India until 2019, and patent protection for erlotinib is the subject of an ongoing court case between Roche and Cipla (see online supplementary appendix 2). Generic erlotinib manufactured by Teva Canada has recently been approved for sale in Canada.^25^ While these basic patents expire in the next 5 years, secondary patents granted on the use of these compounds in combination treatments may pose barriers to generic market entry.

### Global demand

Global demand estimates based on incidence and eligibility are presented in table 3. Erlotinib, sorafenib and lapatinib have considerable volume demand, where even conservative estimates of proportion treated (eg, 30% of eligible population) would yield demands sufficient for sustainable competitive manufacture. For imatinib, estimated volume demands are lower, although still comparable in numbers to, for example, those receiving paediatric second-line HIV treatment.^21^ In the case of imatinib, robust competition is already demonstrated in large export volumes and price reductions seen over the past 5 years.

**Table 3:** Global incidence of indicated cancers, and estimates of total numbers eligible for treatment with selected TKIs

TKI and standard dose	ICD10 category and incidence	Indication of TKI, and percentage of relevant ICD10 group	Eligibility in terms of pathology, and percentage of incident cases with this subtype	Eligibility in terms of stage of disease, a percentage of incident cases at this stage	Total number newly eligible for indication, per year	Total number newly eligible for TKI, per year	Total API requirement per year, in tonnes, to meet incident demand
Imatinib 400 mg QD	Leukaemia (C91–95), 351 965	Chronic myeloid leukaemia, 12.3%	Philadelphia chromosome positive, 87.5%	NA, 100%	37 880	47 999	7.0
	Leukaemia (C91–95), 351 965	Acute lymphoblastic leukaemia, 11.5%	Philadelphia chromosome positive, 25%	NA, 100%	10 119		
Erlotinib 150 mg QD for NSCLC, 100 mg QD for pancreatic cancer	Trachea, bronchus and lung (C33–34), 1 824 701	Non-small cell lung cancer, 85%	Proportion of patients for whom EGFR status can be evaluated and are EGFR positive, 14.6%	Advanced/metastatic, 83.5%	189 082	442 486	19.6
	Pancreatic cancer, 337 872	Pancreatic cancer, 100%	All, 100%	Advanced/metastatic, 75%	253 404		
Sorafenib 400 mg BID	Kidney cancer, 337 860	Renal cell carcinoma, 85%	All, 100%	Advanced/metastatic, 71.5%	205 334	443 734	129.6
	Liver cancer, 782 451	Hepatocellular carcinoma, 87.5%	All, 100%	Advanced/metastatic, 30%	205 393		
	Thyroid cancer, 298 102	Thyroid carcinoma, 95%	Iodine-refractory, 66.6%	Advanced/metastatic, 17.5%	33 007		
Lapatinib 1500 mg QD	Breast cancer, 1 671 149	Breast cancer, 100%	HER-2 positive, 12.5%	Advanced/metastatic, 33.5%	69 979	69 979	38.3

Gastrointestinal stromal tumour, for which imatinib and sunitinib are indicated treatments in some cases, has not been included, due to its relative rarity, and the fact that it spans multiple ICD10 categories. References for figures used in this table can be found in online supplementary appendix 4.

API, active pharmaceutical ingredient; BID, two times a day; EGFR, epidermal growth factor receptor; ICD, International Classification of Diseases; NA, not applicable; NSCLC, non-small cell lung cancer; QD, daily; TKI, tyrosine kinase inhibitor.

### Current prices

**Figure 3:**
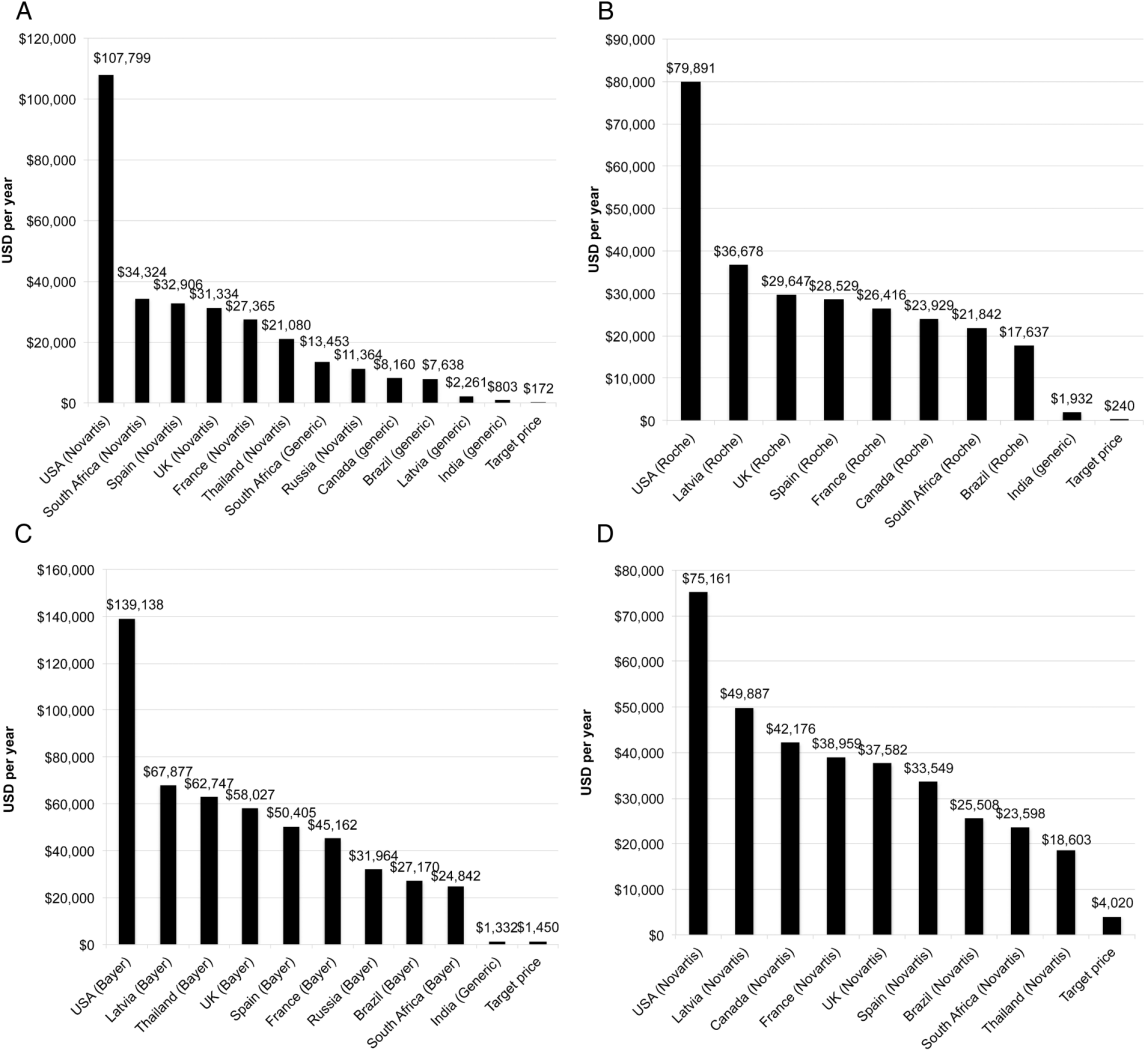
Lowest available prices in selected countries. Lowest available price for (A) imatinib (400 mg) (sources in online supplementary appendix 3); (B) erlotinib (150 mg) (sources in online supplementary appendix 3); (C) sorafenib (400 mg two times a day) (sources in online supplementary appendix 3); and (D) lapatinib (1500 mg) (sources in online supplementary appendix 3) in selected countries.

Figure 3A–D illustrates the ranges across countries in prices for each of the four TKIs analysed. Data sources for these prices are given in online supplementary appendix 3. Indian generic prices (when available) were always found to be significantly lower than all other prices. US prices were, in most cases, at least twice as high as those in EU. There was little variation between brand prices for France, the UK, Spain and, in general, Thai, Brazilian, Russian and South African prices were lower than those of the European countries, with the notable exceptions of sorafenib in Thailand. The lowest price in figure 3C is offered by Cipla, and this is below our target price.^26^

Generic imatinib was available in Canada, Latvia, South Africa, Brazil and India, but not other countries surveyed. Generic erlotinib and sorafenib versions were available in India but not other countries surveyed. Generic versions of lapatinib were not available in any of the countries surveyed.

## Discussion

If produced generically with adequate competition, imatinib, erlotinib, lapatinib and sorafenib can be made available at low prices, making their use feasible in developing countries, and allowing large savings in high-income countries. We demonstrate that generic versions of imatinib can be sustainably and profitably produced at a price between $128 and $216 per person-year, which are far lower than the current prices of around $30 000 in EU and $107 799 per person-year in the USA. Generic erlotinib could be produced for $240 per person-year, versus the current EU prices of $26 416–$36 678, and US price of $79 891. Generic versions of lapatinib and sorafenib can be sustainably produced at 1–11% of the current prices in high-income countries. At the target prices identified, $1 billion would be enough to treat all 1 million patients worldwide who become eligible for treatment with imatinib, erlotinib, sorafenib and lapatinib, every year. This combined cost is less than a quarter of the net sales of $4.7 billion for imatinib in 2013 alone.^27^

The estimates presented in this paper are based on actual, completed sales of API. We assume an inefficient manufacturing process and include all real-world expenses, such as packaging, shipping and duties. Limitations of our analysis include the potential delaying effect of secondary patents. All four drugs analysed are under multiple secondary patents, but the significance of these will not be known until the basic (composition of matter) patents have expired and the existing patents are ‘tested’ by generic companies entering the market. For full cost analyses, other factors would need to be included, such as any additional treatments administered alongside these medicines, the cost of diagnostics, and national health financing mechanisms.

The TKIs surveyed are effective treatments that can be taken orally, are easy to transport and store, and seldom require an advanced care unit. Following lessons learnt from HIV, affordable cancer medication could offer an opportunity to rapidly scale up the treatment in resource-poor settings if combined with infrastructure development and health professional training. In countries where they are under patent protection, cancer medicines at these target prices are likely to become available only after patent expiry. Alternatively, central patents could be invalidated, or compulsory licenses could be issued before patent expiry, as was the case for sorafenib and imatinib in India (see online supplementary appendix 2). In countries where the medicines are not under patent protection, large buyers, such as governments, NGOs and international agencies should encourage the achievement of prices at the levels of our estimates by ensuring that there is effective competition. One option for pharmaceutical companies wishing to increase access to their product without compromising intellectual property rights could be to issue voluntary licenses, such as those for HIV medicines issued to the Medicines Patent Pool.^28^ Our estimates can also inform tenders for medicines and negotiations with current manufacturers. This may be especially relevant to settings where it is not feasible to offer widespread surgical treatment, radiotherapy or traditional chemotherapy. International agencies are investigating options for treatment scale-up. Imatinib was recently included in the WHO Essential Medicines List;^9^ the potential for low prices demonstrated here could allow more cancer medicines to follow. As the medicines surveyed are approaching patent expiry (table 2), generic manufacturers can already begin preparing to launch generic versions, and national and international purchasers can prepare for scaling up of cancer treatment. The price-lowering effects of generic competition have been demonstrated in antiretrovirals for HIV,^20^ where price reductions in excess of 95% have allowed massive increases in the proportion of infected people that are on treatment.

In many cases, decisions on drug indications, their scope and treatment lengths, are based partially on their price. If generic versions are made available at these target prices, this may allow re-evaluation of indication scope, greater duration of treatment, and even combination of TKIs (eg, erlotinib and lapatinib are currently in trials for combination treatments).^29^ Other drugs in the same class as those analysed, such as ibrutinib and vemurafenib, are under patent protection and currently priced at a level that is unaffordable in many settings. Similar analyses may be done for these medicines and other novel cancer treatments.

## Conclusions

Pharmaceutical companies need to recoup investments in research and development to remain financially viable. However, the TKIs analysed have already accumulated billions of dollars in sales, and after patent protection has lapsed, there is no justification for prices to remain significantly above the target prices of production described in this paper. In the case of sorafenib, the CEO of the originator company, Bayer, has commented that the profits made from sorafenib in India do not affect their business model.^30^ The current global prices of TKIs make these treatments unaffordable and unavailable in developing countries and some high-income countries. The findings of this paper demonstrate that scaling up cancer treatment using cheap, generic TKIs is feasible as soon as patent protection is lost. In the interim, alternative mechanisms can be used to reduce prices and allow access to cancer treatments. These mechanisms include using so-called ‘flexibilities’ in the World Trade Organization's Agreement on Trade-Related Aspects of Intellectual Property Rights (TRIPS) to allow generic manufacture and/or importation, and the granting of licenses by originator companies to generic manufacturers, for supply of the developing country market.
